# Association between Parenting Styles and Adolescents’ Mental Disorders: Findings among Pre-University Students

**DOI:** 10.21315/mjms2024.31.3.11

**Published:** 2024-06-27

**Authors:** Siti Roshaidai Mohd Arifin, Nur Syila Syahida Syaziman, Khadijah Hasanah Abang Abdullah, Karimah Hanim Abd Aziz, Khairi Che Mat, Noor Azimah Muhammad, Shanti Wardaningsih

**Affiliations:** 1 Department of Special Care Nursing, Kulliyyah of Nursing, International Islamic University Malaysia, Selangor, Malaysia; 2 National Heart Institute, Kuala Lumpur, Malaysia; 3 Faculty of Medicine and Health Sciences, Universiti Sains Islam Malaysia, Negeri Sembilan, Malaysia; 4 Department of Community Medicine, Kulliyyah of Medicine, International Islamic University Malaysia, Selangor, Malaysia; 5 Psychological Medicine Unit, Faculty of Medicine, Universiti Sultan Zainal Abidin, Terengganu, Malaysia; 6 Department of Family Medicine, Faculty of Medicine, Universiti Kebangsaan Malaysia, Kuala Lumpur, Malaysia; 7 Department of Mental Health Nursing, School of Nursing, Universitas Muhammadiyah Yogyakarta, Indonesia

**Keywords:** adolescent, depression, anxiety, parenting styles, mental health

## Abstract

**Background:**

Existing research indicated a high prevalence of mental health issues among adolescents. Gender and parenting styles are two factors that may influence adolescents’ mental health. Nonetheless, most published studies focused on either secondary school or university students. In contrast, there is a dearth of similar research involving pre-university students. This study aimed to determine the prevalence of mental disorders among pre-university students and their association with parenting styles.

**Methods:**

A cross-sectional study via online questionnaire survey was conducted among students from a pre-university college on the East Coast of Malaysia. Convenience sampling was used to recruit the participants. The questionnaire consisted of three parts: i) sociodemographic data, ii) the Parental Authority Questionnaire and Depression, and iii) the Anxiety and Stress Scale (DASS-21). An online invitation to answer the questionnaire was done via the Student Representative Council (SRC). Data were analysed using descriptive statistics and Pearson’s chi-square test.

**Results:**

A total of 431 participants responded to the online survey. The prevalence of depression, anxiety and stress was 49.0% (*n* = 210), 68.0% (*n* = 293) and 37.6% (*n* = 162), respectively. In addition, father’s educational level (χ^2^ = 10.332, *P* = 0.001) and the authoritarian parenting style (χ^2^ = 10.099, *P* = 0.006) were significantly associated with mental health disorders among adolescents.

**Conclusion:**

The prevalence of mental disorders among pre-university students is relatively high. Pre-university admission mental health screening is vital for early detection and intervention of mental disorders among this vulnerable group. Further research is imperative to establish a comprehensive plan of action that targets parental involvement in managing adolescent mental health disorders.

## Introduction

Mental health disorder is a major public health problem across the world. It encompasses a wide range of disorders that are generally characterised by a combination of abnormal thoughts, perceptions, emotions, behaviours and relationships with others (1). Common mental disorders include depression, anxiety and stress. Any age group of the population can be affected by mental health disorders, including adolescents aged 10 years old–19 years old. At this stage of development, adolescents face a major transition from childhood to adulthood. The transition entails various physical, psychological and socio-emotional challenges, thus making them susceptible to a greater risk of mental health disorders (2, 3). As reported in the literature, mental health disorders such as depression, are one of the major disease burdens and leading causes of morbidity among adolescents globally (1).

If timely diagnosis and proper treatment are not instituted, depression can lead to many adverse impacts, including self-harm and suicide (3). A worldwide mental health survey reported that one-fifth (20.3%) of pre-university students suffer from mental disorders, such as depression, anxiety and substance abuse (4). Furthermore, approximately one-third (31%) of pre-university students aged 18 and above have admitted to having suicidal thoughts (5). Specifically, those who had experienced more than five stressful events in 12 months were more likely to develop suicidal ideation compared to those who did not experience such events (6).

In Malaysia, the prevalence of mental disorders among adolescents showed a rising trend from 10.7% in 1996 to 29.2% in 2015 (7), among which adolescents between 16 years old and 19 years old reported the highest prevalence (34.7%). According to the National Health Morbidity Survey (NHMS) 2017, one in five adolescents in Malaysia had experienced depression (8). Similarly, other local studies also indicated a prevalence of depressive symptoms among adolescents in Malaysia that ranged between 26.2% and 67.8%. Moreover, depression among adolescents was found to be significantly correlated with suicidal thoughts (9–12). According to Ghazali and Azhar (13), among 30 students with depressive symptoms, 13 of them confessed to having the thought of committing suicide, another 12 had suicidal ideation but did not make plans about it, and only 5 did not have any suicidal thoughts. Therefore, the high rates of suicidal thoughts among those suffering from depression is a worrying trend.

Additionally, certain sociodemographic factors are shown to predispose to mental disorders among adolescents. For example, a study among 232 adolescents in Malaysia found that males scored higher on the suicidal ideation scale. In contrast, female adolescents reported a higher rate of depression, anxiety and stress (12). Moreover, adolescents from lower socioeconomic backgrounds were more likely to experience mental health problems (5). Some studies also emphasised the influence of parenting styles in the development of mental health problems among adolescents (14–16). Parenting styles refer to the techniques that parents apply in the upbringing of their children, i.e. the way parents react to their children and how they exert their demands on the children (14). Harsh parenting styles have been shown to cast a negative impact on the mental health of children during their growth. Examples of harsh parenting styles include behaviours of screaming, cursing, threatening and physical punishment to the kids. In contrast, positive parenting encompasses parental warm and nurturing styles that focus on close involvement featuring praise, expressed affection, time commitment and shared positive affect.

Generally speaking, parenting styles can be divided into three categories, namely: i) permissive, ii) authoritarian and iii) authoritative (17). Permissive parents are non-controlling individuals who allow their children to make their own decisions and they are also lenient in punishment. On the other hand, authoritarian parents can be highly directive and controlling, whereby they apply severe punishment to control their children’s behaviour. Lastly, authoritative parents fall somewhere between permissive and authoritative parenting styles. These parents provide clear and firm direction for their children. Disciplinary clarity is exhibited via warmth, reason, flexibility and verbal give-and-take (18). Based on the literature, authoritarian parenting is associated with lower self-esteem among adolescents, while authoritative and permissive parenting help in boosting self-confidence (14, 19).

To date, the majority of the existing literature highlighted a substantial burden of mental disorders among adolescents. However, there is a scarcity of studies involving pre-university students in Malaysia. Thus, this study aimed to determine the prevalence of mental disorders among pre-university students and their association with parenting styles.

## Methods

The study was conducted among 3,000 students aged 18 years old–19 years old who attended a pre-university college located on the East Coast of Malaysia. This college provides foundation programmes for Sciences and Arts streams of studies to prepare candidates for admission into the bachelor’s degree programmes. Data were collected between 16 November 2020 and 7 December 2020. The sample size was calculated based on the single proportion formula in which *n* = (Z/E)2 p(1−p), where Z = 1.96, E = 5% and p = 35%. To account for non-response, the sample size was inflated by 60% to a total of 500. Convenience sampling was applied. The inclusion criteria were: i) students who were registered and enrolled in the foundation programme, ii) those living on campus and (iii) those who stayed with parents before the pre-university programme. Students on study leave were excluded from this study.

Prior to data collection, ethical approval was obtained from the IIUM Research. Upon receiving the ethical approval, the Student Representative Council (SRC) at the college was then approached to obtain a list of student names with their email addresses and contact numbers. The questionnaire was distributed to all participants using a Google Form via their email address or WhatsApp. Participant information sheet and informed consent form were attached in the same Google form. The completed questionnaires would then be submitted as a Google Form to the researcher. The participants could contact the researcher for any inquiries or problems.

### Research Tool

The questionnaires consisted of three parts. Part A captured the sociodemographic data including the gender, race, type of study stream, number of siblings, household income, as well as the marital status, education level and employment status of the parents. Part B consisted of the Parenting Authority Questionnaire (PAQ) to measure parental authority from the perspective of the children who are older adolescents and young adults (17). It includes 30 items on three subscales: i) permissive (P: items 1, 6, 10, 13, 14, 17, 19, 21, 24 and 28); ii) authoritarian (A: items 2, 3, 7, 9, 12, 16, 18, 25, 26 and 29); and iii) authoritative/flexible (F: items 4, 5, 8, 11, 15, 20, 22, 23, 27 and 30). The items were evaluated based on a Likert scale ranging from 1 = strongly disagree to 5 = strongly agree, giving the total sum of 10–50 for each subscale. The parenting style is determined by the subscale with the highest score. The validity and reliability of the PAQ were confirmed in a previous study, with Cronbach’s alpha values of 0.618 for permissive parenting style, 0.733 for authoritarian parenting style and 0.738 for authoritative parenting style (17, 19), respectively.

Lastly, Part C of the questionnaire was made up of the Depression, Anxiety and Stress Scale (DASS-21). While it cannot be used to diagnose mental health disorders, DASS-21 is commonly used to screen the prevalence of depression, anxiety and stress among the respondents. The questionnaire consists of 21 items on a Likert scale of 0–3 (0 = did not apply to me at all; 1 = applied to me to some degree or some of the time; 2 = applied to me to a considerable degree or a good part of the time and 3 = applied to me very much or most of the time). The Malay version of the DASS-21 (20) has been validated in a previous study. It was shown to have good reliability based on Cronbach’s alpha value of 0.81 (depression), 0.89 (anxiety) and 0.78 (stress). It also has excellent internal consistency as well as good discriminative, concurrent and convergent validities (21).

### Data Analysis

Data were analysed using IBM SPSS version 26.0. All results were described as frequencies and percentages. The prevalence of mental disorders (depression, anxiety and stress) was expressed using descriptive analysis. The independent variables were the perceived parenting style and sociodemographic data. The term ‘perceived’ was used since the information was gathered from the children’s perspective and not the parental point of view. The dependent variable was the status of mental disorders. Pearson’s chi-square test was performed to identify the factors associated with depression, anxiety and stress. All statistical significance was taken as a *P*-value of less than 0.05.

## Results

The questionnaire was distributed to 540 students, of which 431 completed the questionnaire, indicating a response rate of 81%. Table 1 shows the baseline characteristics of the study participants. More than half of them were females (65.9%, *n* = 284). They were almost equally distributed between the Science stream (55.0%, *n* = 237) and the Arts stream (45.0%, *n* = 194). As high as 88.9% (*n* = 383) of them had three or more siblings while the remaining 11.1% (*n* = 48) were from families of one or two siblings.

In terms of household income, 40.1% (*n* = 173) of the study participants came from families with monthly incomes of less than RM5,000 while more than half (59.9%, *n* = 258) were from families with monthly incomes of more than RM5,000. As for the marital status, the majority (89.1%, *n* = 384) of the participants’ parents were still married. Most of their fathers (61.5%, *n* = 265) and mothers (61.0%, *n* = 263) were of tertiary education levels. As for employment status, more students had mothers (37.8%, *n* = 163) who were retired or unemployed as compared to fathers (20.2%, *n* = 87).

**Figure 1:**
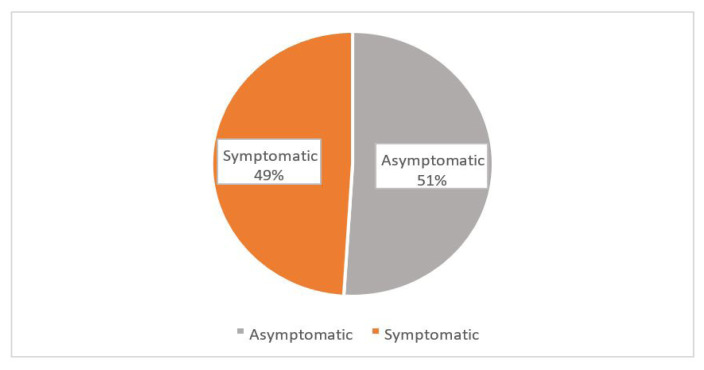
The prevalence of depression among the study participants

**Figure 2:**
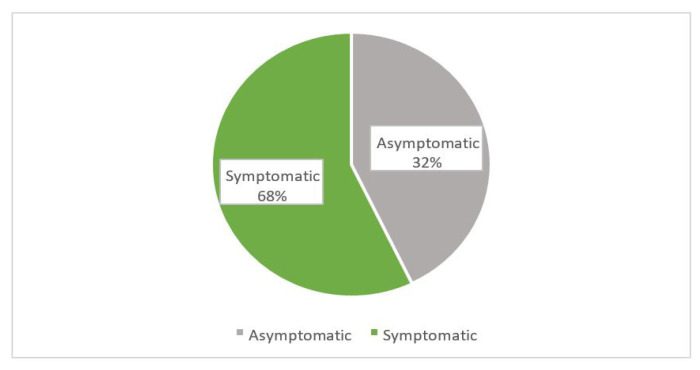
The prevalence of anxiety among the study participants

**Figure 3:**
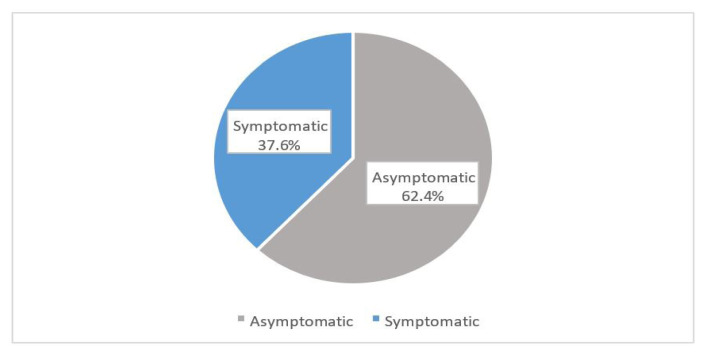
The prevalence of stress among the study participants

Figure 1 shows the prevalence of depression among pre-university students. Nearly half of the students (49.0%, *n* = 210) were symptomatic while (51.0%, *n* = 221) were asymptomatic. Based on Figure 2, the prevalence of anxiety was the highest (68.0%, *n* = 293) among the three mental health problems. Figure 3 shows that the majority of the students (62.4%, *n* = 269) reported a normal level of stress and only (37.6%, *n* = 162) students reported symptoms of stress. No association was found between sociodemographic factors with depression or stress among the study participants. However, anxiety was found to be associated with fathers’ education level (χ^2^ = 10.332, *P* = 0.001). As shown in Table 2, the remaining variables did not show any significant association with mental disorders.

As illustrated in Table 3, there was an association between parenting styles and depression (χ^2^ = 10.099, *P* = 0.006). Among students who were symptomatic of depression, the majority were identified as having authoritarian parents (64.6%, *n* = 53), followed by permissive (47.2%, *n* = 34) and authoritative (44.8%, *n* = 124) parents. From Table 4, no association was observed between parenting styles and anxiety among pre-university students. Most of the symptomatic students had authoritative parents (68.6%, *n* = 190), followed by authoritarian (70.7%, *n* = 58) and permissive (62.5%, *n* = 45) parents. Lastly, there was also no association between parenting styles and stress among pre-university students (Table 5). Most of the non-stress students had authoritative parents (*n* = 277), followed by authoritarian (*n* = 82) and permissive (*n* = 72) parents.

## Discussion

This study aimed to determine the prevalence and factors associated with mental health disorders among pre-university students using DASS-21 screening. The findings revealed a high prevalence of anxiety among the students, similar to a study conducted in Malaysia in which pre-university students reported a relatively high level of anxiety (22). The prevalence of anxiety is believed to be linked to stressors such as financial constraints, remote online learning, as well as uncertainty of future events related to academics and career. Furthermore, as this study was conducted during the COVID-19 pandemic, the students could have been predisposed to a higher level of stressors, thus worsening their anxiety.

In this study, the prevalence of depression was 49.0%, twice higher than the 21.4% worldwide prevalence reported among 13,984 college students across eight countries in a previous study (21.2%) (4). We postulated that the higher prevalence of depression during the study period coincided with the COVID-19 pandemic. This was supported by another study in France that also reported a higher prevalence of depression among 69,054 university students aged 18 and above who were surveyed using an online questionnaire during the pandemic (23). The French study also recorded a rise of 16.1% in the prevalence of depression among the same group of participants with severe depression compared to the previous year. During the pandemic, all the students were confined at home to undergo online learning as they were not allowed to stay on campus. As a result, social isolation and poor peer support might have contributed to the development of depressed emotions. Additionally, a study conducted among public university students in New York suggested that poor financial conditions as well as COVID-19-related factors such as social distancing and remote online learning were the main stressors that induced a high prevalence of depression (22).

Apart from that, the study results were also consistent with previous research that highlighted the significant association between parents’ educational level and the development of mental disorders among children (24, 25). In this study, the father’s education affected the prevalence of anxiety among pre-university students. Similarly, a previous study stated that the higher the parents’ education level, the less likely their children are to get depression, anxiety and stress (24, 25). This may be due to a better understanding of the risks of diseases and techniques for improving health among parents with higher education levels. Moreover, parents with high education are usually associated with more stable emotional health as they are more adept at practising positive coping strategies in addressing stressors. As a result, parents with good educational background are more likely to produce children with good mental health (26).

Next, this study also found an association between parenting styles and the prevalence of depression. The finding is congruent with a previous study conducted among 5,216 private university students in Malaysia (27). The study revealed that university students raised by permissive and authoritarian parents had a higher tendency to experience depression, anxiety and stress. Permissive parents commonly practise a hands-off parenting style, allowing their children to make their own decision and deal with emotional struggles on their own. In contrast, the authoritarian parenting style can come across as too strict until the children choose to internalise their emotional problems.

While this study contributed to the understanding of adolescents’ mental health and its associated factors, there are some limitations. First of all, this was a cross-sectional study design whereby the exposure and the outcome were concomitantly assessed, thus making it impossible to establish the causal effects (28). Furthermore, the convenience sampling method restricts the generalisation of the results to other pre-university students or other general populations. Besides, information on the participants’ previous educational background was not obtained. The secondary-level education system in Malaysia is made up of boarding schools and day schools. A previous study among 493 Chinese adolescents showed that students from boarding schools had higher emotional intelligence and resilience (29). Students from boarding schools might develop stronger peer support compared to their counterparts in non-boarding schools, thus making them less susceptible to depression, anxiety and stress.

## Conclusion

In view of the increasing prevalence of mental health crises among adolescents in Malaysia, this was a timely study to determine the prevalence and contributing factors of mental disorders among pre-university students. Addressing its objective to determine the prevalence of mental disorders among pre-university students, this study indicated that the prevalence of anxiety and depression among the students was high. Therefore, we recommend that it is vital to perform mental health screening before university admission to facilitate early detection and intervention for the students. In addition, early prevention and intervention programs should be implemented to encourage help-seeking behaviours to minimise suicidal ideations and attempts (30). On top of these strategies, periodical monitoring by counsellors and the formation of online support groups can be instituted as preventive measures to reduce the prevalence of mental disorders. This study also aimed to assess the association between parenting styles and adolescents’ mental disorders. We have found that parenting styles play a vital role in the development of adolescents’ mental health disorders. Thus, further research to develop an appropriate plan of action that targets parents’ involvement in managing mental health disorders among adolescents is required. In the long term, the protection of the mental wellbeing of pre-university students can ensure their mental wellbeing and academic success.

## Figures and Tables

**Table 1:** Background characteristics of the study participants

	Total (*n*)	
Gender		
Male	147	34%
Female	284	66%
Type of stream		
Arts	194	45%
Science	237	55%
Number of sibling		
Less than 3 siblings	48	11%
3 and more siblings	383	89%
Household income		
< RM5,000	173	40%
> RM5,000	258	60%
Parent’s marital status		
Separated/Divorced/Widowed	47	11%
Married	384	89%
Father’s education level		
Primary/Secondary education	166	39%
Tertiary education	265	61%
Mother’s education level		
Primary/Secondary education	168	39%
Tertiary education	263	61%
Father’s employment status		
Unemployed/Retiree	87	20%
Employed	344	80%
Mother’s employment status		
Unemployed/Retiree	163	38%
Employed	268	62%

**Table 2:** Association between sociodemographic characteristics with anxiety symptoms

	Anxiety % (*n*)		Total (*n*)	Value (χ^2^)	*P*-value

	Asymptomatic	Symptomatic			
Gender					
Male	34.0 (50)	66.0 (97)	147	0.408	0.523
Female	31.0 (88)	69.0 (196)	284		
Type of stream					
Arts	32.5 (63)	67.5 (131)	194	0.034	0.854
Science	31.6 (75)	67.4 (162)	237		
Number of sibling					
Less than 3 siblings	37.5 (18)	62.5 (30)	48	0.746	0.388
3 and more siblings	31.3 (120)	68.7 (263)	383		
Household income					
< RM5,000	28.3 (49)	71.7 (124)	173	1.813	0.178
> RM5,000	34.5 (89)	65.5 (169)	258		
Parent’s marital status					
Separated/Divorced/Widowed	25.5 (12)	74.5 (35)	47	1.02	0.313
Married	32.8 (126)	67.2 (258)	384		
Father’s education level					
Primary/Secondary education	22.9 (38)	77.1 (128)	166	10.332	0.001*
Tertiary education	37.7 (100)	62.3 (165)	265		
Mother’s education level					
Primary/Secondary education	28.0 (47)	72.0 (121)	168	2.067	0.151
Tertiary education	34.6 (91)	65.4 (172)	263		
Father’s employment status					
Unemployed/Retiree	27.6 (24)	72.4 (63)	87	0.984	0.321
Employed	33.1 (114)	66.9 (230)	344		
Mother’s employment status					
Unemployed/Retiree	31.9 (52)	68.1 (111)	163	0.002	0.968
Employed	31.1 (86)	67.9 (182)	268		

Note:

* *P*-value < 0.05

**Table 3:** The association between parenting styles with depressive symptoms

Variables	Depression % (*n*)		Total (*n*)	Value (c^2^)	*P*-value

	Asymptomatic	Symptomatic			
Parenting style					
Authoritarian	35.4 (29)	64.6 (53)	82	10.099	0.006*
Authoritative	55.2 (153)	44.8 (124)	277		
Permissive	52.8 (38)	47.2 (34)	72		

Note:

* *P*-value < 0.05

**Table 4:** The association between parenting styles with anxiety symptoms

Variables	Anxiety % (*n*)		Total (*n*)	Value (c^2^)	*P*-value

	Asymptomatic	Symptomatic			
Parenting style					
Authoritarian	29.3 (24)	70.7 (58)	82	1.326	0.515
Authoritative	31.4 (87)	68.6 (190)	277		
Permissive	37.5 (27)	62.5 (45)	72		

**Table 5:** The association between parenting styles with stress symptoms

Variables	Stress % (*n*)		Total (*n*)	Value (χ^2^)	*P*-value

	Asymptomatic	Symptomatic			
Parenting style					
Authoritarian	54.9 (45)	45.1 (37)	82	4.477	0.107
Authoritative	66.1 (183)	33.9 (94)	277		
Permissive	56.9 (41)	43.1 (31)	72		
